# Impact of cervical cancer on quality of life: beyond the short term (Results from a single institution): Quality of life in long-term cervical cancer survivors: results from a single institution

**DOI:** 10.1186/s40661-015-0011-4

**Published:** 2015-09-19

**Authors:** J. Khalil, S. Bellefqih, N. Sahli, M. Afif, H. Elkacemi, S. Elmajjaoui, T. Kebdani, N. Benjaafar

**Affiliations:** National cancer Institute, Mohamed V University, Rabat, Morocco

**Keywords:** Long term cervical cancer survival, Quality of life, Sexual functioning, QLQ-C30, QLQ-CX24, Population based study

## Abstract

**Background:**

Cervical cancer (CC) is one of the most widespread gynecological malignancies in women worldwide. Treatment strategies and screening modalities have largely evolved these past years resulting in an improvement of survival. However, treatment modalities are associated with long term side effects that significantly impacts quality of life (QOL) in cervical cancer survivors. The aim of this study is to evaluate QOL (General and sexual QOL) in cervical cancer survivors up to 10 years after the diagnosis.

**Material and methods:**

In a cross-sectional descriptive study design, 110 cervical cancer survivors (CCS) and 80 healthy controls completed questionnaires assessing QOL.

**Results:**

Participants were Arabic White, sexually active. The mean age at diagnosis was 34 years and was 43 years at the time of the interview. In our series long term CCS have generally a good global QOL comparable with healthy controls. However, issues concerning emotional functioning were over expressed by CCS. As to the sexual impact of cervical cancer; CCS experienced less sexual functioning and enjoyment and less satisfaction with their body image when compared to healthy controls.

In a multivariate analysis, spiritual well-being and social support were the predictor factors that statistically affected QOL among the studied cohort, it accounted for 81 % of the variance in QOL scores.

**Conclusions:**

A better understanding of the complexity of the relationship between QOL and cervical cancer sequelae in one hand and socio-demographic factors in the other hand is necessary to improve QOL among cervical cancer survivors. More efforts should make to inform disease free patients about expected side effects and symptoms to face the physical changes that would affect their QOL and sexual activity.

## Headline

Survivorship relationships between QOL and sequelae of cervical cancer for women diagnosed during fertile age are complex. It is important for health care professionals to recognize that aspects of cancer survivorship require attention and specific follow-up care.

## Background

Cervical cancer (CC) is one of the most widespread gynecological malignancies in women worldwide [1]. In Morocco, CC is the second most common cancer with an estimated 640 new cases in 2008 only in the radiation oncology department of the national cancer institute.

Treatment strategies and screening modalities have largely evolved these past years; consequently cervical cancer is diagnosed at earlier stages where treatment is more effective. Overall survival rates have in fact widely increased; in the earlier stages 5-year relative survival is estimated to be around 91.5 % [2].

With therapeutic progress come side effects; either it is surgery, radiotherapy or chemotherapy literature has widely described toxicities and side effects related to the treatment adopted. Persistent negative effect on physical and psychosocial functioning of the treatment especially concurrent chemoradiotherapy have also been described in many studies [3–5].

After completion of treatment, cervical cancer survivors (CCS) have to deal with treatment sequelae which eventually lead to a deterioration of their quality of life (QoL). Moreover, cervical cancer is mostly diagnosed during the fertile age where the majority of patients are sexually active [6]. Sexual activity in addition to self esteem is also highly influenced by the treatment received. Therefore, discussions and planning of primary treatment should address all of the QoL aspects.

In Morocco, unfortunately few studies and surveys have addressed this question especially the sexuality issues after cervical cancer, it is mostly due to religious beliefs and mores that make this subject a taboo for most women.

Our study is aiming to analyze the impact of cervical cancer on QOL and sexuality among long term cervical cancer survivors.

## Materials and methods

### Study design and recruitment

Our study is a prospective one. Case eligibility included women sexually active, diagnosed with cervical cancer 5–10 years earlier, aged between 17 and 45 at the time of diagnosis and free of any recurrences or second malignancies.

Women were identified during routine follow up.

The study was properly explained to eligible participants. After obtaining a written informed consent, the study questionnaire was administered during 30 min by physicians using appropriate words adapted to our patients mostly illiterates. For patients intimacy all interviews were conducted by female physicians.

### Participants

#### Cases

Of the180 women recruited in this study, 70 patients declined and 110 accepted to participate in the study.

Of the 110 patients included in the study, 55 completed the questionnaire in writing and provided signed informed consent, the remaining patients provided their answers verbally and the questionnaire was filled by the physician in charge, their consent was provided either verbally by 15 women or by fingerprinting in 40 cases.

#### Controls

To evaluate the results of CCS, random controls were selected; eighty unaffected acquaintance controls completed the questionnaire.

Controls were mostly the accompanying persons of the patients. Although we were unable to match at least one control per case, the ascertained controls provide valuable information regarding the used scales.

### Instruments

#### Quality of life

Quality of life was evaluated using the Arabic validated version of the European Organization for Research and Treatment of Cancer (EORTC) QOL Questionnaire, QLQ-C30, which is a 30-item questionnaire assessing the general QOL of cancer patients. It measures global health, 5 functional domains (physical, role, cognitive, emotional,and social), 3 symptom scales (fatigue, pain, and nausea/vomiting), and 6 symptom or problem single-item scales (dyspnea, insomnia, appetite loss, constipation, diarrhea, and financial problems) [7, 8].

#### Sexual impact

Sexual impact on cervical cancer survivors (CCSs) was evaluated using specific questionnaire elaborated and validated by the EORTC (QLQ-CX24) that concerned only CCSs. It contains three multi-item scales (symptom experience, body image, and sexual/vaginal functioning) and six single-item scales (lymphedema, peripheral neuropathy, menopausal symptoms, sexual worry, sexual activity, and sexual enjoyment) [9].

Since the Arabic version is still not available in the EORTC database we proceeded to a translation from English to Arabic language. We used a method involving translation and back-translation procedures, in collaboration with professionals, psycho-oncologists and a translator/ linguist. A pilot test was performed. We asked a random sample of 20 patients was studied to participate in the study. All of them accepted and completed the questionnaire. All of the patients and their accompanying female’s persons found the questionnaire straightforward and easy to complete.

In addition to the aforementioned questionnaires, we collected information concerning family, social and professional status, comorbidity, education level and marital status, number of children, life insurance and employment. Information regarding tumor stage and treatment received were also required for our data collection.

#### Spiritual well-being

Spiritual well-being was measured by the Functional Assessment of Cancer Therapy-Spirituality Scale (FACT-Sp) [10]. It is 12-item scale with three sub-domains of spiritual well-being, which permit an in-depth exploration of the components that constitute spiritual well-being (peace, meaning, and faith).

This scale was mainly used to evaluate the spiritual well being as a predictor factors affecting QOL in CCS.

### Data collection and procedure

Routine follow up is performed in a dedicated unit to gyneco-mammary malignancies; questionnaires were administered to the participants in this special unit right after physical exam. Because of the high prevalence of illiteracy, we could not proceed with a self-administration; the questionnaires were explained properly by radiation oncologists to each patient. All of the participants completed the questionnaires. Ultimately, 55 patients completed the questionnaire in writing while 50 % of the cohort provided their answers verbally and the questionnaires were filled by the physician in charge.

## Statistical analysis

The data were stored and analyzed using SPSS version 17.0 software (SPSS Inc., Chicago, IL, USA).

Univariate analysis (Pearson product moment correlations) was used to determine the relationships between the variables collected such as age, education level, tumor stage. To account for multivariate relation of the predictor variables to QOL, a stepwise multiple regression was performed.

In order to identify variables significantly linked to QOL scores, we performed a multivariate analysis. The alpha level was set at PV: 0.05 to determine statistical significance.

## Results

### Demographic and clinical characteristics

**Table 1:** Demographic characteristics of the studied cohort

	Studied cohort	Control arm
Mean age at diagnosis	34 years (range 17–43)	
Mean age at the time of the interview	43 years (range 25–53)	42 years (range 23–51)
Marital status:		
Married	100 %(*n* = 110)	100 %(*n* = 80)
Children		
Yes	91 % (*n* = 100)	93 %(*n* = 74)
No	9 % (*n* = 10)	7 % (*n* = 6)
Number of children		
< 3	34 %(*n* = 37)	26 % (*n* = 21)
> 3	66 %(*n* = 73)	74 % (*n* = 79)
Educational level		
Illiterate	56 %(*n* = 61)	58 %(*n* = 46)
Elementary	14 %(*n* = 15)	11.6 %(*n* = 9)
Junior college	18 %(*n* = 20)	15.4 %(*n* = 13)
High school	13 %(*n* = 14)	15 %(*n* = 12)
Employment status:		
Employed	28 %(*n* = 31)	20 %(*n* = 16)
Unemployed	72 %(*n* = 79)	80 %(*n* = 40)
Life Insurance:		
Yes	89 %(*n* = 98)	Not needed
No	11 %(*n* = 12)	
Hospitalization during the last year:		
Yes	8 %(*n* = 9)	Not needed
No	92 %(*n* = 101)	
Social support		
Yes	76 %(*n* = 84)	Not needed
No	24 %(*n* = 26)

**Table 2:** Clinical characteristics of cervical cancer survivors

	Percent	Number
Tumor stage:		
IB	4 %	4
IIA	14 %	15
IIB	15 %	17
IIIA	20 %	22
IIIB	47 %	52
Treatment modalities:		
Surgery	21 %	23
*Surgery only*	4 %	4
*Surgery + Radiotherapy*	10 %	11
*Surgery + Radiotherapy + Chemotherapy*	7 %	8
Concurrent chemoradiotherapy	79 %	87
Brachytherapy	67 %	74

The majority of the participants were married, Arabic white, mostly illiterates in 56 % of the cases and unemployed in 72 % of the cases. The mean age at diagnosis was 34 years (range 17–43) and 43 years (range 25–53) at the time of the interview.

The average time between the year of diagnosis and year of interview was 7.0 years (range: 4–11 years).

Tumor reported stages were IB in 4 % of the cases, IIA, IIB, IIIA and IIIB in 14, 15, 20 and 47 % of the studied cohort. Treatment modalities included concurrent radiochemotherapy in most of the cases (79 %), surgery was performed in 21 % of the cohort; surgery only (4 %); surgery and radiation (10 %); surgery, radiation, and chemotherapy (7 %).

Eighty nine percent of the studied cohort had a life insurance covering the whole treatment process. (Demographc and clinical characteristics are detailed in Tables 1 and 2).

### QOL scores of the studied cohort (Table 3)

**Table 3:** The EORTC quality of life questionnaire (QLQC30) for the studied cohort and controls

QLQ C 30	Patients	Controls	*P* value
Global quality of life	71.07 ± 21.5	74.8 ± 19.8	0.24
Functional scales, Mean ± SD			
Physical functioning	78.1 ± 25.6	71.07 ± 21.5	0.15
Role functioning	76.8 ± 26.8	79.8 ± 24.6	0.45
Emotional functioning	75.8 ± 37.5	86.9 ± 29.7	0.001
Cognitive functioning	73.7 ± 25.6	84.7 ± 24.6	0.24
Social functioning	93.6 ± 21.5	91.5 ± 19.3	0.07
Symptom scales, Mean ± SD			
Energy/Fatigue	15.6 ± 18.2	12.3 ± 8.4	0.26
Nausea and vomiting	2.9 ± 12.6	1.9 ± 5.6	0.15
Pain	21.1 ± 27.8	12.5 ± 6.7	0.49
Short of breath	8.4 ± 20.8	2.3 ± 5.8	0.43
Sleep disturbance	17.2 ± 31.3	8.5 ± 5.6	0.3
Lack of appetite	9.8 ± 20.3	7.6 ± 10.4	0.23
Constipation	20.5 ± 39.8	12.3 ± 22.8	0.016
Diarrhea	13.7 ± 39.3	5.6 ± 11.3	0.23
Financial problems	35.3 ± 38.2	27 ± 28.9	0.1

*P value relates to the comparison with the control arm

QOL was assessed using the EORTC QLQ-C30 questionnaire. As indicated in Table 3, cervical cancer survivors generally reported good global QOL, it was particularly evident among the social, physical and role functioning domains.

On the symptoms scales CCS showed a relatively low level of problems, the most reported symptom was pain, financial problems were also highly reported; although concerning financial problems, we cannot conclude if cancer is the main cause mostly because the majority of the included patients had already a low socioeconomic level.

FACT-SP scale was also used to assess general QOL among cervical cancer survivors. We did not find statistical difference between the studied cohort and the control arm, participants generally expressed a good spiritual well being reflected in the three sub domains (peace, meaning, and faith) of the scale used, only 17 % of the studied cohort expressed low scores regarding the peacefulness sub domain.

### Comparison of QOL scores between cervical cancer survivors and controls (Table 4)

**Table 4:** Quality of life scores of cervical cancer survivors and controls (Significant adjusted differences)

	CCS	Controls	*P*
Global quality of life	71.07 ± 21.5	71.07 ± 21.5	0.15
Functional scales			
Emotional functioning	75.8 ± 37.5	86.9 ± 29.7	0.001
Symptoms scales			
Constipation	20.5 ± 39.8	12.3 ± 22.8	0.016

Only few differences were noted between controls and long term cervical survivors included in the study. Compared with controls, CCS reported mostly a lower emotional functioning on the QLQ-C30 scale (*p* = 0.0) and a higher rate of constipation.

## Sexual functioning

Of the 110 cervical cancer survivors interviewed, 77 (61.6 %) were sexually active at the time of the interview. Multiple reasons were reported by patients not sexually active; 31 % of them reported having no interest in sexual relations, 36 % did not have sexual partner, the fear of developing a relapse or an infection was reported by 41 % of the cohort, fatigue, physical problems and other reasons were reported in 10, 13 and 8 %.

Some scales of the EORTC QLQ-CX24 questionnaire had missing values because of variability concerning patient’s acceptance. Eventually, scales concerning Sexual/Vaginal Functioning and Sexual Enjoyment were skipped by 12 and 14 % of the studied cohort. Scales regarding sexual worry and sexual activity had also missing values; 9 and 8 % of the women did not answer these questions.

**Fig. 1:**
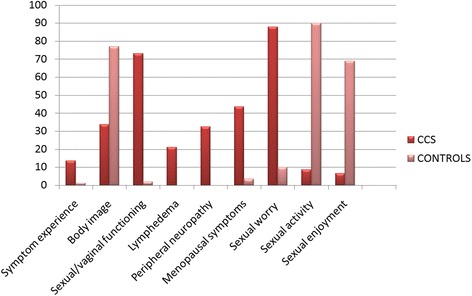
Disease-specific quality of life scores (EORTC QLQ-CX24) of cervical cancer survivors compared with controls

When compared to the control arm CCS experienced more lymphedema (P = 0.02), less sexual functioning and enjoyment and less satisfaction with their body image. Figure 1 illustrates differences concerning sexual functioning between the two arms.

## Predictors of quality of life

The resulting model of QOL outcomes suggests that spiritual well-being significantly affects QOL in CCS. Spiritual WB accounted for 81 % of the variance in QOL scores of the sample.

We also analyzed demographic and clinical characteristics to assess variables affecting QOL in CCS. In a univariate analysis tumor stage and the treatment received were defined as predictors of QOL; in fact advanced tumor stage, brachytherapy and the absence of social support were associated with lower scores of QOL-C30 and QOL-CX24 suggesting a poorer health related QOL in CCS. In the multivariate analysis only social support and spiritual WB were defined as predictors of QOL.

## Discussion

These past years, oncologists have focused their efforts on maximizing the overall survival of cervical cancer patients. Even if there is a general acknowledgement that QOL is an important aspect of patient care, it is not the main priority when recommending cancer treatment.

There is an increasing interest to evaluate and improve QOL among CCS. In fact, many population-based studies have been enrolled to assess QOL among CCS and to define factors affecting patient’s well being after the diagnosis of cervical cancer.

To our knowledge, our series is the first population-based study to assess QOL in long term Moroccan CCS up to 10 years after cancer treatment.

The particularity of our series is that it concerns Arabic female patients for whom sexuality is considered as a taboo not commonly talked about; also QOL among cancer survivors is still not a priority.

In our study we characterized QOL issues of young, long-term female cervical cancer survivors with a Moroccan and also a Muslim perspective, thereby adding to the literature a description of a group of women who has not previously been studied.

As for most published data [5, 11–13], in our study health related QOL in long term CCS was generally satisfactory ; we did not find a statistical difference between the studied cohort and healthy controls. Nevertheless, emotional functioning was lower in CCS.

As anticipated, we could not assess correctly sexual functioning of our patients; most of them expressed no interest in having sexual activity, also many of the sexually active patients skipped some items in the questionnaire relative to sexual functioning. For whom who completed the questionnaire, sexual functioning was mostly compromised due to physical discomfort, in fact cervical cancer survivors reported more discomfort than controls with a higher incidence of hot flashes and vaginal dryness.

As a result of our study, it is quite obvious that difference in sexual functioning between CCS and healthy controls remains till 10 years after the diagnosis of cervical cancer. Suggesting that cervical cancer survivors continue to struggle with physical problems and discomfort which negatively affect their sexual functioning and gynecologic health. These results are consistent with those of other published studies [5, 14, 15]. Therefore, gynecological care and sexual education after cervical cancer treatment should an important component of the initial treatment.

In fact, available guidelines strongly recommend sexual education in CCS, they also suggest the use of dilators 2–4 weeks after the completion of Radiotherapy treatment [16, 17]. In a recent review evaluating different interventions used for psychosexual dysfunction in women treated for gynaecological malignancy, oestrogen vaginal therapy appeared to improve vaginal dryness [18].

Unfortunately in our daily practice sexual education is still not a priority for many physicians, so are dilatators that were not prescribed in any of the studied cases. Of note, local hormonal therapy was prescribed in only 20 % of the cases with vaginal dryness.

We tried to find an explanation to the lack of the discussion about sexual issues in CCS in our department, for that we asked physicians why sexual education was not performed and we gave them multiple choices of answers, sample answers were 1/Not feeling comfortable talking about sexuality with patients, 2/Patients are not interested, 3/ It is not a priority, 4/ Lack of time, the second and the third choices were the most represented (47 and 52 % respectively).

Many predictive factors for QOL in CCS have been studied and published in the literature [5, 14, 15], as a example Wenzel et al. found that spiritual well-being, maladaptive coping, and reproductive concerns were independently affecting QOL [5], other studies suggest that social, family, and intimate relationship played a crucial role on perception of QOL [19].

In our series, spiritual well being along with social support significantly affected QOL in CCS.

Although we could not define a cause-and-effect relationship, it is reasonable to hypothesize that CCS who experience changes in their marital status after the diagnosis of cervical cancer, are more likely to have lower QOL scores. In our study, 39 % of CCS who were married when diagnosed got divorced at the time of the interview. Seventy two percent of them attributed their divorce to cervical cancer. This suggests an additional area for investigation regarding the stress that this disease causes to the relationship.

Our study showed that the treatment modalities used did not statistically affect health-related QoL or sexual functioning, which is in contrast with other reports where surgery was associated with less sexual dysfunction and better QOL when compared to radiation therapy [11, 20–24]. In our opinion, it is due to the fact that only 4 % of the studied cohort had surgery alone as the main treatment, making the statistical analysis not feasible.

This study has several limitations, in one hand only two available scales were used to assess QOL(QLQ-C30,QLQ-CX24) as Arabic validated version is still not available for other scales such as SF36, Coping Orientations to Problems Experienced (COPE) scale, the Interpersonal Support Evaluation List scale (ISEL)and Likert scale for reproductive concerns, therefore we could not define all predictor factors for QOL. On the other hand, although our efforts to complete the QOL-CX24 relative to sexual functioning, obtaining full acceptance of all the patients was not achieved which made the evaluation of sexual functioning incomplete.

## Conclusion

Cervical cancer survivors are dealing each day with sequelae relative to their disease. A better understanding of long-term QOL in cervical cancer survivors seems to be crucial, especially that it will allow physicians to inform patients about what they could expect in a long term. A literature review has in fact suggested that a well informed patient deals better with treatment sequelae [23]. Improving patient-doctor relationship, counseling about cancer specific issues and sexual education should be the major objectives in the initial patient’s care.

## Consent

Informed consent was obtained from all the patients of the publication.
